# Soil moisture enhances the vibrations induced by moving earthworms, especially of detritivores

**DOI:** 10.1098/rsos.260667

**Published:** 2026-10-21

**Authors:** Renée Veenstra, Wouter Halfwerk, Mohamed Henriques, Jurgen van Hal, Matty P. Berg, Theunis Piersma

**Affiliations:** ^1^ Conservation Ecology Group, Groningen Institute for Evolutionary Life Sciences (GELIFES), University of Groningen, Nijenborgh 7, 9747 AG Groningen, The Netherlands; ^2^ BirdEyes, Centre for Global Ecological Change at the Faculties of Science & Engineering and Campus Fryslân, University of Groningen, Wirdumerdijk 34, 8911 CE Leeuwarden, The Netherlands; ^3^ Amsterdam Institute for Life and Environment, Section Ecology and Evolution, Vrije Universiteit Amsterdam, De Boelelaan 1107, 1081 HV Amsterdam, The Netherlands; ^4^ Science Team Sustainability & Wellbeing, Faculty Campus Fryslân, University of Groningen, Doelestrjitte 8, 8911 DX Leeuwarden, The Netherlands; ^5^ Department of Coastal Systems, NIOZ Royal Netherlands Institute for Sea Research, Landsdiep 4, 1797 SZ 't Horntje, Texel, The Netherlands

**Keywords:** agricultural soils, remote-sensing predators, acoustic emissions

## Abstract

Earthworms (Lumbricidae) are moisture-loving soil-dwellers that can be categorized into two feeding guilds with distinct behaviours: detritivores and geophages. Detritivores feed on litter fragments and geophages on smaller litter particles mixed with soil. When earthworms move within and across soil layers to forage, soil characteristics might influence the scale of these movements and their detectability to predators. To investigate the extent to which the detection of earthworm movements and the vibrations they thereby generate is affected by soil moisture, we used an innovative method to detect and quantify vibrations of moving earthworms emitted through the soil. We designed a mesocosm experiment in which we recorded, with a geophone, the movement-induced vibrations of detritivores and geophages in drier and wetter soils. Earthworm vibrations were detected best at 200 Hz and were detected significantly more in wetter than in drier soils. Moreover, in wetter soils, vibrations induced by detritivores were detected more frequently and at higher intensities than those of geophages. These findings suggest differences in activity levels between detritivores and geophages, as a function of soil moisture content. The observed activity patterns are likely to have food web consequences because earthworm consumers relying on mechanoreceptors to detect prey might detect detritivores more often, specifically in wetter soils.

## 1. Introduction

Earthworms (Lumbricidae) are soft-bodied, predominantly subterranean organisms with a wide geographical distribution range [1]. They can be grouped in three ecological categories—anecic, epigeic and endogeic earthworms (although a more nuanced functional group categorization based on bioturbation behaviour also exists [2])—or two feeding guilds: detritivores and geophages [3,4]. Detritivores, on one hand, mainly eat undecomposed organic matter, such as partly fragmented dead plant material, which they gather on or near the soil surface and transport below ground [5,6]. Geophages, on the other hand, feed on mineralized, small-sized organic matter particles mixed with soil and, therefore, consume more soil than detritivores [7,8]. In general, anecic (e.g. *Lumbricus terrestris*) and epigeic species (e.g. *Lumbricus rubellus*) belong to the detritivores, while endogeic species (e.g. *Aporrectodea caliginosa*) are geophages [2–4]. Through their feeding activities, earthworms affect many soil processes and functions, such as soil formation, soil aeration and water infiltration, organic matter decomposition and nutrient cycling [9,10].

In addition to their importance for soils, earthworms also serve as prey for many predator species [11,12]. The subterranean movements of earthworms that perform activities, like foraging, can indicate their presence, and for their consumers relying on mechanoreceptors it can provide cues for their detection [13,14]. Nevertheless, earthworm detection in the soil is not only determined by burrowing activity but also by soil type and soil moisture content, as vibrations propagate more easily through wetter than drier soils [15–17]. For these reasons, activity levels of detritivores and geophages under different soil moisture conditions are consequential, not only for soil services but also for predators' detection capacities. However, as earthworms are hidden to the human eye, movement activity levels as a function of soil moisture content are not easy to measure.

Multiple methods have been applied to investigate the subterranean activity of earthworms. For instance, earthworm burrowing behaviour can be described using radio-labelled earthworms [18,19], X-ray tomography [20] or video recordings of earthworms moving in transparent ‘soil’ [21]. In addition, underground movements of earthworms have been observed by recording movement-induced vibrations [15] or acoustic emissions (AE), using a variety of recording devices like an AE monitoring system [22] or audio loggers [23]. In addition to assessing the soil functions provided by earthworms, such methods are also informative for investigating the effect of their movements on detection by predators. Hence, this study used a geophone to record vibrations to measure the movement activity of buried earthworms, information that is likely to be relevant for earthworm predators sensitive to prey vibrations, such as moles or shorebirds [10,13].

Because water content is an important abiotic soil variable that can affect both earthworm activities [24] as well as the transmission of vibrations produced when earthworms move [25], we designed a mesocosm experiment to investigate the extent at which the vibrations emitted by detritivore and geophage earthworms are detectable through the developed methodology, and how vibration detectability varied between the feeding guilds and the soils with different moisture contents. To achieve this, we first compared soil columns with and without earthworms to (i) determine the frequency at which earthworm vibrations were best detected and (ii) to test the effect of soil moisture level (dry versus wet soils) on earthworm vibration detection. Second, focusing only on soil columns with earthworms, we (iii) compared the vibrations generated by detritivore and geophage earthworms, within both dry and wet soils. The recorded vibrations were expressed as two different variables: threshold crossings (an indication of earthworm movement detection frequency) and vibration intensity (indicating the intensity or strength of the vibrations produced by earthworm movement).

## 2. Methods

### 2.1. Experiment preparations

In November 2024, we collected soil in southwest Friesland, the Netherlands. We collected 12 soil cores of 20 × 20 × 20 cm from a single grassland used for dairy farming (N 52°59′45, E 5°26′18), with a sandy clay loam soil, among the most common soil types in this area [26]. We took the soil cores to the laboratory of the A-LIFE Ecology & Evolution section of the Vrije Universiteit Amsterdam for further processing. There, we hand-sorted the samples to remove all the macrofauna, vegetation and roots, as well as larger sediment organic matter fragments.

To measure the effect of soil moisture content on earthworm vibration detection, we divided the soil into two batches to create two treatment levels: wet and dry soils. Each batch was first mixed thoroughly by hand to re-homogenize the soil. One of the batches remained untreated (hereafter referred to as batch 1), representing the ‘wet soil’ treatment (see §2.2). We determined the moisture content of batch 1 by taking a subsample of the soil, which was weighed and dried for 48 h at 105°C in an oven, and then re-weighed to obtain the dry mass (to the nearest 0.01 g). Afterwards, the soil moisture percentage was calculated using the following formula:

((wet mass − dry mass)/dry mass)×100.

Batch 1 had a final soil moisture content of 67%. Batch 2 (treated) was oven-dried at 30°C until it reached a soil moisture content between 40 and 50%. Both during and after the drying process, the soil was repeatedly homogenized. We aimed for this moisture content range, since it provided a noticeable contrast with the wet soil treatment (approx. 20%), while not inducing drought stress for earthworms [27,28]. Ultimately, batch 2 had a soil moisture content of 44% and was used for the ‘dry soil’ treatment. We placed both batches in a sealed box to prevent re- or dehydration before or during the experiment and stored them at 21°C, the temperature at which we performed the experiment.

Also in November 2024, we collected and hand-sorted earthworms from the grassland soils of a dairy farm near Foxwolde, Drenthe, the Netherlands (N 53°09′42, E 6°28′09). These parcels have a sandy loam soil [29], which contains less clay and more sand particles than the soil used in the experiment. However, the earthworm species collected are common throughout the Netherlands and occur in a diverse range of soil types [30], so we are confident that this did not have a relevant effect on the experimental results. In the laboratory, the earthworms were pooled into six groups for each earthworm feeding guild (i.e. detritivores and geophages). Each experimental group consisted of 20 individuals with an average fresh biomass of 13.38 g (range: 12.77–14.18 g) for detritivores and 13.43 g (range: 12.65–14.23 g) for geophages. Old and young earthworm individuals were divided equally across all groups, which ensured a very similar size distribution in each experimental group for both feeding guilds. Further species and age-class identification revealed that detritivores consisted of adult and juvenile *L. rubellus* and juvenile *L. terrestris*, and geophages consisted of adult and juvenile *A. caliginosa*. All three species are very common in large parts of Europe and are considered invasive on other continents [1].

To build soil columns in which we measured vibrations, we used PVC tubes with a closed bottom plate, measuring 19.5 × 16.5 cm (height × diameter). To create standardized and homogeneous soil bulk densities, we added soil in layers of 2 cm and, after each layer, compacted the soil using a tamping device with a mass of 7 kg that we set carefully on top of the soil for 3 s. This process was repeated until the tube was filled with 10 cm of soil. After all the measurements in a soil column replicate were taken, we quantified the soil bulk density and moisture content of that column replicate. These values showed that, at the end of the experiment, the moisture content of the soils had not significantly changed and a clear distinction between soil moisture treatments (dry: 42–44% and wet: 64–67%) remained. Moreover, the mean soil bulk density for dry and wet soils was also very similar (0.742 g cm^–3^ versus 0.734 g cm^–3^, respectively).

### 2.2. Experimental design and soil vibration recordings

To measure soil vibrations, we implemented a stratified experimental set-up involving a total of 36 soil columns and three treatments: earthworm presence treatment (columns with versus without earthworms), soil moisture treatment (columns with dry soil versus wet soil) and earthworm feeding guild treatment (columns with detritivore earthworms versus geophage earthworms). These treatments were grouped into six ‘treatment groups’, each with six column replicates: (i) dry soil with detritivore earthworms, (ii) wet soil with detritivore earthworms, (iii) dry soil with geophage earthworms, (iv) wet soil with geophage earthworms, (v) dry soil without earthworms, and (vi) wet soil without earthworms. We first compared columns with and without earthworms to (i) determine whether and at which frequencies our recording set-up was able to pick up relevant vibratory cues, and to (ii) test the effect of soil moisture on earthworm vibration detections. Next, we used only columns with earthworms to (iii) test the differences in vibrations between soil columns with different earthworm feeding guilds.

To reduce external disturbances as much as possible, we carried out measurements in the basement of the laboratory building and placed soil columns in an 80 × 80 × 80 cm metal box covered with sound and vibration-damping material (ArmaSound). Furthermore, the box was placed on a 10 cm layer of additional damping material (Metisse). To control for strong variation in remaining background noise from the building, we conducted the experiment in six rounds, in which we sequentially measured columns across treatments. Each round started with filling six columns (three with dry soil and three with wet soil), which we placed in the vibration-damping box at randomly chosen positions. Earthworms were placed on top of the soil in their assigned columns in a fixed order, always starting with the column positioned in the upper left-hand corner and moving towards the column in the lower right-hand corner. Once the earthworms had disappeared below the soil surface, we added 2 g of vegetation (grasses/roots) to provide them with sufficient organic material during the measurements. The time between adding the earthworms to the columns and the first measurement was approximately 20 minutes for each column.

During each round (which lasted for approx. 2 h), we recorded soil vibrations for 1 minute in each of the six columns. We used an omnidirectional geophone (Xeophone, Tremor Labs; 10–1000 Hz; 80 V m^–1^ s^–1^), connected to a Soundbook (Sinus MK2, eight-channel measurement system) to record vibrations using the program SAMURAI (version 3.0, base software plus FFT analyser). We placed the 5 cm long pin of the geophone in the centre of each soil column. A geophone integrates waves propagating along three-dimensional axes and can be used to compare relative differences in vibrations, as well as changes in patterns and spectral content of these vibrations. We also made use of a vertical velocity meter (Sinus ICP seismometer, 10–315 Hz range, sensitivity 29 V m^–1^ s^–1^), in the first place for calibration by attaching it to the geophone in the vertical as well as horizontal directions and by recording the voltage signals from both sensors simultaneously on the Soundbook. Based on this calibration session, we set the recording values for the geophone at 100 V m^–1^ s^–1^ in our recording program. We also made recordings of background noise vibrations along the vertical axis with the velocimetre, which were in the range of –120 to –160 dB (m s^–1^) between the 20 and 315 Hz frequency band.

Data were stored by the program in 1/3 octave bands, starting at 10 Hz, up to 1000 Hz, for every 0.02 seconds. To quantify the background building vibrations, each measurement of a column with earthworms was immediately (10–20 s) followed by a measurement of a column without earthworms with the same soil moisture treatment level. We then compared vibrations in columns with earthworms to those without earthworms (background noise recordings) to assess if we were able to detect earthworm movement (and in which frequency band). This means that we took twice as many measurements in each column without earthworms (10) as in each column with earthworms (five). However, since we have twice as many treatment groups with earthworms (four) as without earthworms (two), this resulted in a balanced number of measurements per treatment group (table 1).

**Table 1.** Summary of sample sizes of the soil column (*N* = 36) and measurements (*N* = 233) replicates for each of the three treatments (earthworm presence, soil moisture and earthworm feeding guild) used in the mesocosm experiment studying the effect of soil moisture and earthworm feeding guild in the detectability of earthworm vibrations. Each soil column with earthworms was measured five times, and each soil column without earthworms (working as controls for the earthworm presence treatment) was measured 10 times. Before statistical analysis, three measurements in columns with dry soil and detritivore earthworms, and three measurements in columns with dry soil without earthworms were removed (see §2.4). In addition, one measurement without earthworms in dry soil was accidentally skipped during the experiment. In each measurement, vibrations were recorded at all frequencies (125, 160, 200, 250 and 315 Hz) at the same time. The experiment involved three treatments: earthworm presence treatment (columns with versus without earthworms), soil moisture treatment (columns with dry soil versus wet soil) and earthworm feeding guild (columns with detritivore earthworms versus geophage earthworms). These treatments were grouped into six ‘treatment groups’, each with six column replicates: (i) dry soil with detritivore earthworms, (ii) wet soil with detritivore earthworms, (iii) dry soil with geophage earthworms, (iv) wet soil with geophage earthworms, (v) dry soil without earthworms (used to quantify background noise), and (vi) wet soil without earthworms.

Research question	Treatment groups	Column replicates	Measurement replicates
(1) Which frequency best distinguishes earthworm vibrations from background vibrations?	with earthworms	24	117
	without earthworms	12	116
(2) How does earthworm vibration detections differ between soil moisture levels?	dry soil with earthworms	12	57
	dry soil without earthworms	6	56
	wet soil with earthworms	12	60
	wet soil without earthworms	6	60
(3) How does vibration detections differ between earthworm feeding guilds?	dry soil with detritivores	6	27
	dry soil with geophages	6	30
	wet soil with detritivores	6	30
	wet soil with geophages	6	30

Between rounds of measurement, we removed the earthworms from the columns and recounted them. We then mixed the used soil thoroughly back with the remainder of the soil and formed six new columns for the next round of measurements. After conducting three rounds of measurements, in which all six groups of each earthworm feeding guild were used, we mixed and redistributed all earthworms to form six new detritivore and geophage groupings. Thus, each column replicate with earthworms housed a unique earthworm group, but individual earthworms were set to perform twice in the experiment. At the end of the experiment, after opening the columns, we found four dead earthworms in four different columns. We still used data from these columns as these dead earthworms were mostly small individuals, and we did not suspect their mortality to have caused biases in our results.

### 2.3. Vibration variables as indications for earthworm movement

To understand how earthworm vibrations could be distinguished from background noise and how variable the vibration readings were, we inspected spectrograms of recordings from columns with and without earthworms (figure 1). Based on these spectrograms, we concluded that the frequency bands between 100–300 Hz showed the highest potential to distinguish earthworm vibrations from background noise. This is because vibrations at higher frequency bands attenuate faster, while those at lower frequencies are more likely to be masked by background noise. Consequently, we continued the preliminary analyses for vibration recordings at five 1/3 octave frequency bands: 125, 160, 200, 250 and 315 Hz.

**Figure 1.**
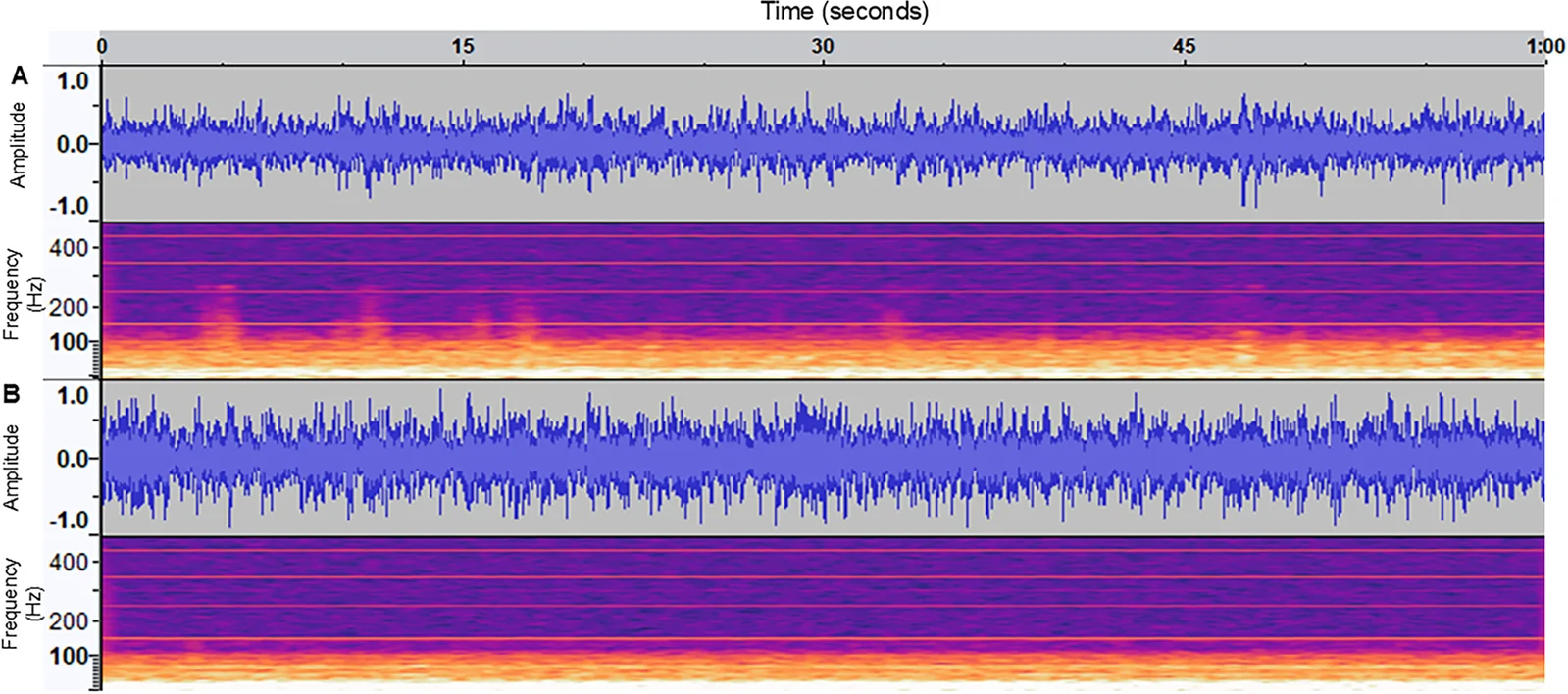
Two example spectrograms (bottom) with amplitude envelopes (top) showing vibrations in soil columns with (A) and without (B) earthworms. The abscissa displays the time in seconds (1:00 being 60 seconds), and for the spectrograms, the ordinate displays the frequency (in Hz), and the colours represent the intensity of the vibrations, with a gradient going from dark blue (lowest) to light-yellow (highest) vibrations.

We calculated the mean and variance of the vibrations recorded in all treatment groups (supplementary material, figs. S1 and S2). These analyses revealed that both the mean and variance of the 1 minute vibration recordings were highly variable within and between columns with and without earthworms, and between frequency bands, probably owing to background noise. Moreover, the mean and variance of the vibrations were higher in dry soils than in wet soils, which is probably due to the weight of the columns, since dry soils have a lower mass than wet soils, in theory resulting in better background vibration propagation. Through these preliminary analyses, we concluded that, especially when comparing earthworm vibrations between dry and wet soils, the mean and variance were not appropriate variables. We, therefore, focused on the detection of specific, short-term vibration events which we termed ‘threshold crossings’ that deviated strongly from the background vibratory levels.

To determine threshold crossings, we counted the number of times that recorded vibration values crossed a certain amplitude threshold. This threshold was determined by measuring the background vibrations in three columns without earthworms in both wet and dry soils. Each of those columns was measured three times during 1 minute, on an evening with little activity in the laboratory building, thus with low background vibration levels. These constituted ‘quiet controls’. We calculated the average variance in background vibration for each frequency band in wet and dry soils. We then set the threshold by taking the median dB of a measurement and adding the averaged variance of the quiet controls, thereby creating a flexible threshold that differs slightly for each measurement, but importantly, was determined based on separate control recordings. Next, we calculated the number of times this predetermined threshold was crossed (see supplementary material, figs. S3 and S4 for examples of a measurement with and without earthworms, respectively). Threshold crossings were considered to be caused either by earthworm movements or by external disturbances. Comparing threshold crossings from columns with and without earthworms allowed us to assess whether threshold crossings of a given soil column were caused by earthworms.

In addition to the number of times vibrations crossed a set threshold, we also analysed the intensity of these events, which we refer to as ‘peak intensity’. To do so, we calculated the mean dB of the highest 1% data points of each measurement, from which we subtracted the mean dB of the total measurement (see supplementary material, figs. S5 and S6 for examples of a measurement with and without earthworms, respectively). This value thus represents the absolute difference between the amplitude ‘peaks’ (highest 1% data points) and the mean dB of the measurement and can provide insight into the relative amplitude of the most intense earthworm vibrations. Our rationale behind this variable is that earthworms that generate vibrations of a higher intensity, e.g. by stronger movements or movements by larger parts of their bodies, may be easier picked up by a predator. By comparing peak intensity values from columns with and without earthworms, we assessed whether earthworm movements generated vibrations that were high enough to exceed background noise and thus potentially be picked up by predators. However, since the peak intensity values of each measurement are dependent on the mean and variance of dB recorded, which can be higher in dry soils than in wet soils for certain frequencies (supplementary material, figs. S1 and S2), peak intensity values were only compared between earthworm feeding guilds within the same moisture level and not between dry and wet soils.

### 2.4. Data analysis

Prior to the statistical analysis, we carefully inspected the data and reviewed the experiment notes to remove errors and unwanted data recordings. Subsequently, we removed three measurements of detritivores in dry soil columns and their following measurements in dry soil columns without earthworms, since we noticed afterwards that not all earthworms had disappeared in the soil yet. Movements on top of the soil showed different waveform patterns than movements within the soil, making these measurements incomparable with the rest of the experiment. Moreover, we removed the first 0.5 s from each recording, as this included a peak generated by pushing the ‘start’ button for the experiment.

All statistical analyses were conducted within R, version 4.2.2 [31]. To investigate at which frequencies earthworm movements within the soil are best detected, we compared threshold crossings and peak intensity of vibrations between columns with and without earthworms using generalized linear mixed models (GLMM), applied through the *glmmTMB* package [32]. We built one model for each response variable: for threshold crossings, we conducted a GLMM with the negative binomial family, and for peak intensity, we used a log-transformed *y* and conducted a Gaussian GLMM. For both models, we used earthworm presence (categorical variable with two levels: with and without earthworms), frequency band (discrete variable with five levels: 125, 160, 200, 250 and 315 Hz) and their interaction, as independent variables (see table 1 for sample sizes). Column replicate number (or: ‘round’ number, as each column replicate was measured in a different ‘round of measurements’, discrete variable with six levels, one through six) was used as a random intercept to account for the fact that soil columns have multiple replicate measurements. We executed Tukey's post hoc tests using the *emmeans* package [33] to make pairwise comparisons between columns with and without earthworms for each frequency band. The frequency band showing the biggest differences was selected to be used in the remaining analyses.

To assess the effect of soil moisture content on the detection of earthworm movement at the selected frequency band (see above), we used GLMMs for each response variable, with earthworm presence (with two levels: with and without earthworms), soil moisture treatment (with two levels: wet and dry) and their interaction as independent variables. To test for differences in earthworm movement detection between earthworm feeding guilds, we removed all columns without earthworms from the dataset and conducted a GLMM, including the earthworm feeding guild treatment (with two levels: geophages and detritivores), soil moisture treatment (wet and dry) and their interaction, as independent variables. Moreover, to exclude the possibility that geophages may be better detected than detritivores at a frequency band other than 200 Hz, we ran a GLMM for both response variables that included earthworm feeding guild, frequency band and their interaction as independent variables. For this model, we only included columns with earthworms in wet soils. All models in this paragraph included the column replicate number as a random intercept. We used a negative binomial GLMM for threshold crossings and a log-transformed Gaussian GLMM for peak intensity. We computed Tukey's post hoc tests to make pairwise comparisons between earthworm feeding guilds only within moisture levels. For all statistical models, the confidence level was set to 95%.

## 3. Results

The threshold crossings in the frequency bands centred around 200 and 250 Hz were the most indicative of earthworm movements (figure 2*a*; tables 2 and 3). For the peak intensity of these vibrations, we found the highest values in columns with earthworms in the 200, 250 and 315 Hz bands (figure 2*b*; tables 2 and 3). For both threshold crossings and peak intensity, the 200 Hz frequency band registered the largest differences between columns with and without earthworms (tables 2 and 3). We therefore used recordings made in this frequency band for the remaining analyses.

**Figure 2.**
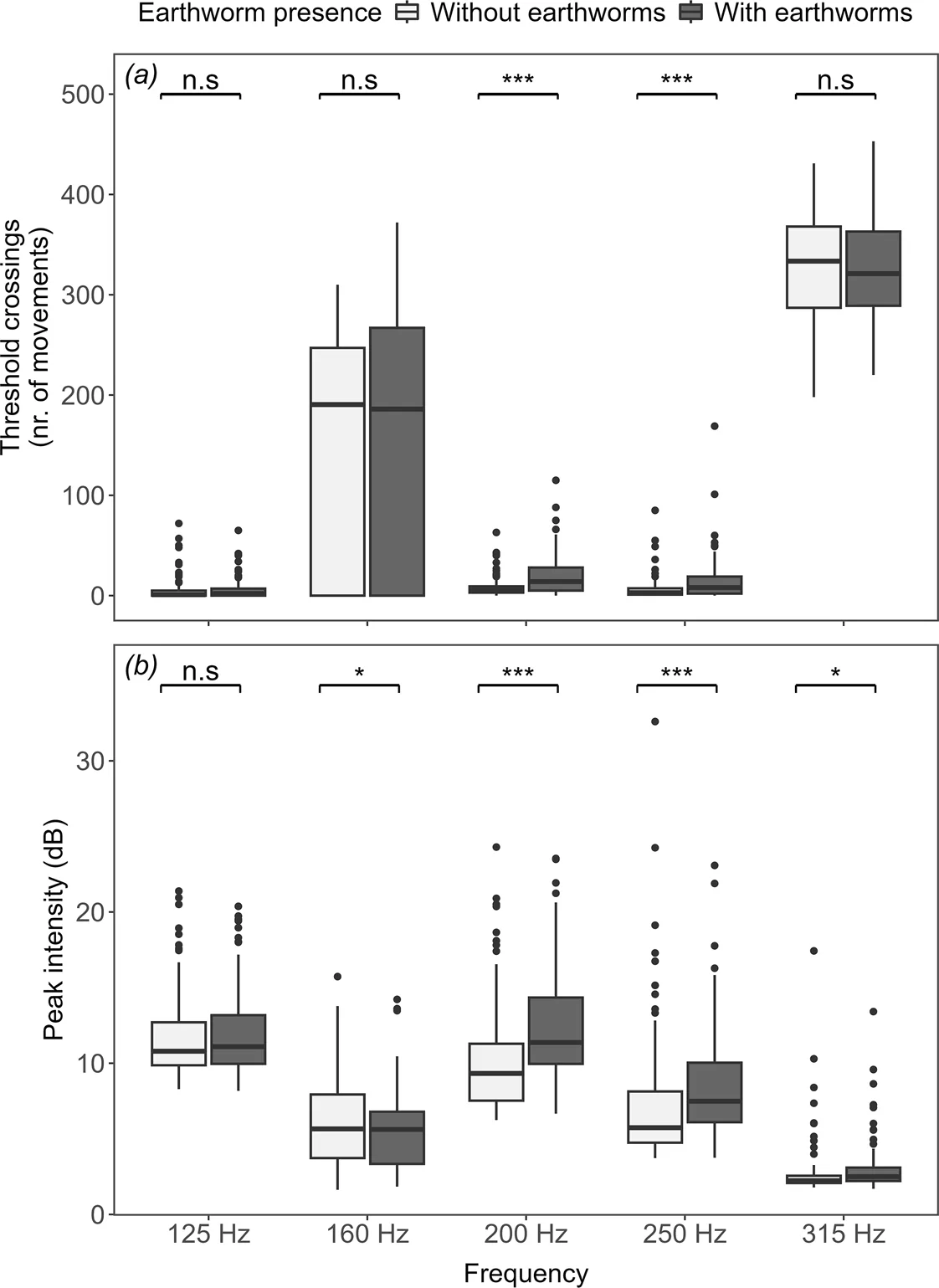
(*a*) Comparisons of number of threshold crossings and (*b*) peak intensity of vibrations between columns with (dark bars) and without (light bars) earthworms, for soil vibration measurements at five different frequency bands (abscissa). The number of threshold crossings indicates the frequency of earthworm movement, whereas the peak intensity of vibrations describes the intensity of earthworm movements. Statistical significances of pairwise comparisons are shown as * above the box plots; non-significant differences are indicated with ‘n.s.’ (see §2; tables 2 and 3 for more information).

**Table 2.** Model result summaries of two GLMMs testing the frequency band at which earthworm movements—threshold crossings and peak intensity—can best be detected (*N* = 1165). The model for threshold crossings included a negative binomial family, whereas the model for peak intensity used a log-transformed Gaussian family. Both models included earthworm presence (with versus without earthworms), frequency band (125, 160, 200, 250 and 315 Hz) and their interaction as fixed effects, and soil column replicate number as a random intercept. For earthworm presence, columns without earthworms are the reference level, and for frequency band, the 125 Hz band is the reference. See §2 for more details.

	estimate	SE	*z-*value	*p-*value
**Threshold crossings**				
Intercept	1.691	0.149	11.272	**< 0.001**
Earthworm presence: With earthworms	0.082	0.193	0.426	0.670
Frequency:				
160 Hz	3.154	0.190	16.563	**< 0.001**
200 Hz	0.461	0.193	2.388	**0.017**
250 Hz	0.128	0.194	0.661	0.509
315 Hz	4.107	0.191	21.547	**< 0.001**
Earthworm presence * Frequency:				
With earthworms: 160 Hz	–0.003	0.267	–0.009	0.993
With earthworms: 200 Hz	0.756	0.270	2.801	**0.005**
With earthworms: 250 Hz	0.730	0.271	2.691	**0.007**
With earthworms: 315 Hz	–0.092	0.267	–0.344	0.731
**Peak intensity**				
Intercept	2.527	0.031	81.770	**<0.001**
Earthworm presence: With earthworms	0.010	0.040	0.250	0.803
Frequency:				
160 Hz	–0.641	0.040	–16.040	**<0.001**
200 Hz	–0.153	0.040	–3.840	**<0.001**
250 Hz	–0.502	0.040	–12.570	**<0.001**
315 Hz	–1.270	0.040	–31.780	**<0.001**
Earthworm presence * Frequency:				
With earthworms: 160 Hz	–0.102	0.056	–1.810	0.070
With earthworms: 200 Hz	0.168	0.056	2.970	**0.003**
With earthworms: 250 Hz	0.143	0.056	2.530	**0.011**
With earthworms: 315 Hz	0.075	0.056	1.340	0.181

**Table 3.** Tukey's post hoc tests with pairwise comparisons of threshold crossings and peak intensity values at five frequency bands between columns with and without earthworms. Post hoc tests were conducted following the statistical models listed in table 2.

	Estimate	SE	*z-*value	*p-*value
**Threshold crossings**				
Without vs. with earthworms				
125 Hz	–0.082	0.193	–0.426	0.670
160 Hz	–0.080	0.184	–0.433	0.665
200 Hz	–0.838	0.188	–4.453	**<0.001**
250 Hz	–0.812	0.190	–4.271	**<0.001**
315 Hz	0.010	0.184	0.052	0.959
**Peak intensity**				
Without vs. With earthworms				
125 Hz	–0.010	0.040	–0.249	0.803
160 Hz	0.093	0.040	2.314	**0.021**
200 Hz	–0.177	0.040	–4.448	**<0.001**
250 Hz	–0.153	0.040	–3.828	**0.001**
315 Hz	–0.085	0.040	–2.139	**0.033**

When assessing the effect of the soil moisture treatment on earthworm movement detection, we found that in wet soils there was a significantly higher number of threshold crossings and the vibration peak intensity values were significantly higher in columns with earthworms compared with columns without earthworms (figure 3; tables 4 and 5). In dry soils, this difference was smaller; there were still significantly more threshold crossings in columns with earthworms, but there were no significant differences between peak intensity values (figure 3; tables 4 and 5). Both earthworm feeding guilds displayed a higher number of threshold crossings in wet soils than in dry soils (figure 4; tables 6 and 7).

**Figure 3.**
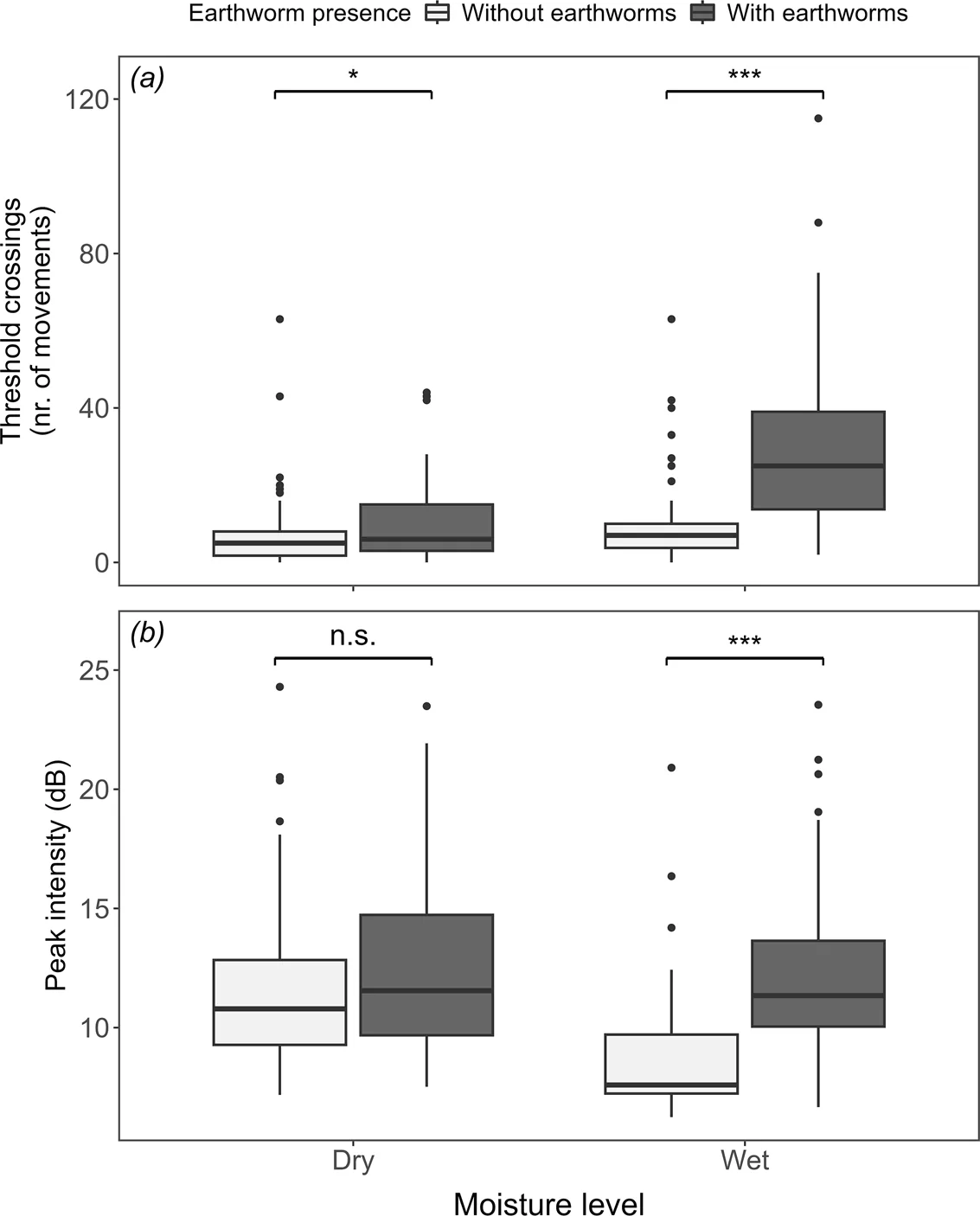
(*a*) Comparisons of the number of threshold crossings and (*b*) peak intensity values between columns with earthworms (dark bars) versus without earthworms (light bars) in dry and wet soils, at the 200 Hz frequency band. The number of threshold crossings indicates the frequency of earthworm movement. The peak intensity of vibrations describes the intensity of earthworm movements. Statistical significances of pairwise comparisons are shown as * above the box plots; non-significant differences are indicated with ‘n.s.’ (see §2; tables 4 and 5 for more information).

**Table 4.** Model result summaries of two GLMM testing the effect of soil moisture treatment on earthworm detection—threshold crossings and peak intensity (*N* = 233). The model for threshold crossings included a negative binomial family, whereas the model for peak intensity a used log-transformed Gaussian family. Both models included earthworm presence (with versus without earthworms), soil moisture treatment (dry versus wet) and their interaction as fixed effects, and soil column replicate number as a random intercept. For earthworm presence, columns without earthworms are the reference level, and for soil moisture treatment, wet soil is the reference. See §2 for more details.

	Estimate	SE	*z-*value	*p-*value
**Threshold crossings**				
Intercept	2.262	0.134	16.925	**<0.001**
Earthworm presence: With earthworms	1.079	0.172	6.257	**<0.001**
Moisture level: Dry	–0.255	0.180	–1.416	0.1567
Earthworm presence: With earthworms * Moisture level: Wet	–0.660	0.251	–2.630	**0.009**
**Peak intensity**				
Intercept	2.245	0.032	69.430	**<0.001**
Earthworm presence: With earthworms	0.296	0.046	6.470	**<0.001**
Moisture level: Dry	0.267	0.047	5.740	**<0.001**
Earthworm presence: With earthworms * Moisture level: Wet	–0.246	0.066	–3.740	**<0.001**

**Table 5.** Tukey's post hoc tests with pairwise comparisons of threshold crossings and peak intensity values between columns with and without earthworms, in dry and wet soils. Post hoc tests were conducted following the statistical models listed in table 4.

	Estimate	SE	*z-*value	*p-*value
**Threshold crossings**				
*Without vs. with earthworms*				
Moisture level: Dry	–0.418	0.182	–2.295	**0.022**
Moisture level: Wet	–1.079	0.172	–6.257	**<0.001**
**Peak intensity**				
*Without vs. With earthworms*				
Moisture level: Dry	–0.050	0.047	–1.065	0.288
Moisture level: Wet	–0.296	0.046	–6.470	**<0.001**

**Figure 4.**
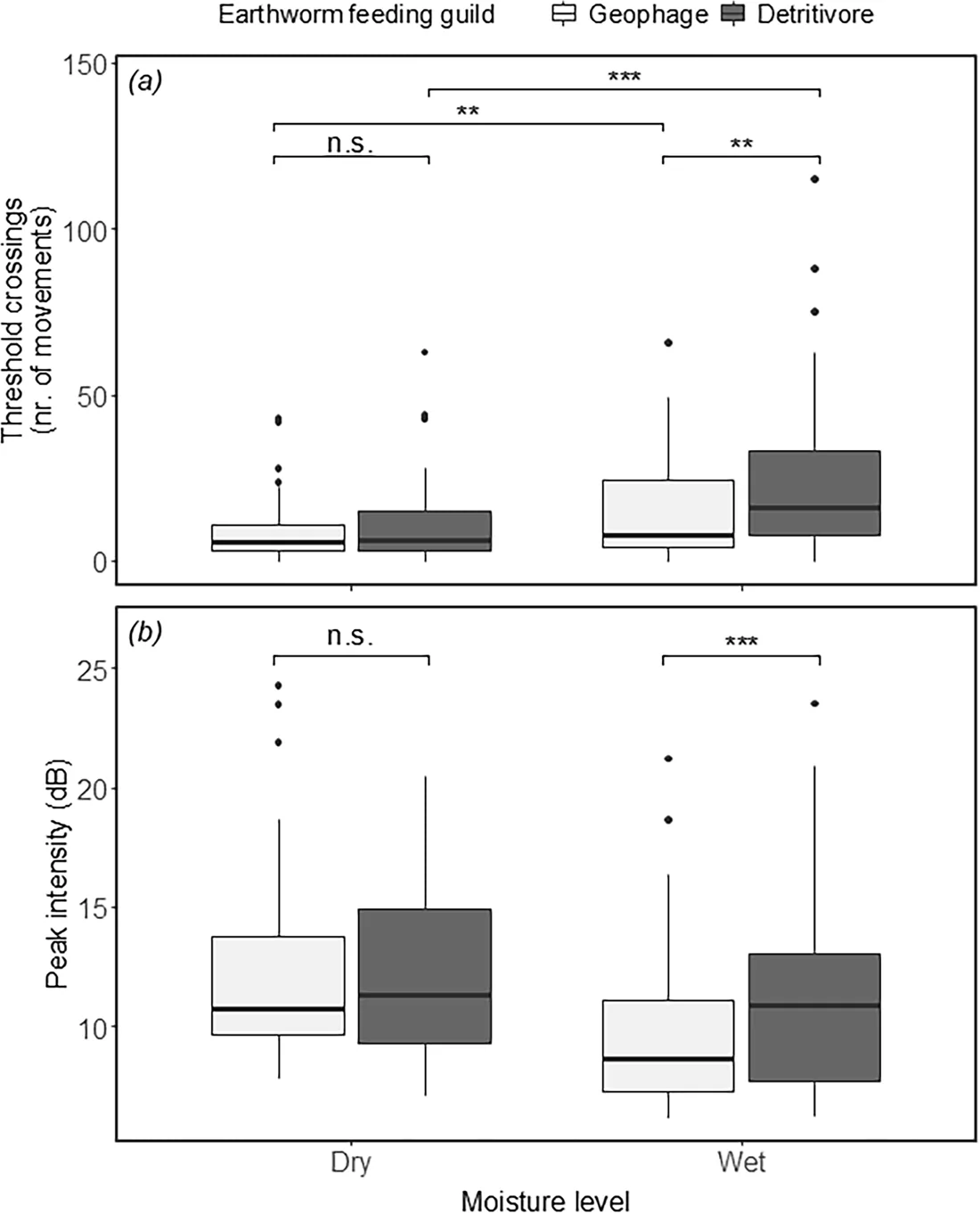
(*a*) Comparisons of the number of threshold crossings and (*b*) peak intensity values between geophages (light bars) and detritivore (dark bars) earthworms in dry and wet soils, at the 200 Hz frequency band. The number of threshold crossings indicates the frequency of earthworm movement. The peak intensity of vibrations describes the intensity of earthworm movements. Statistical significance of pairwise comparisons are shown as * above the box plots; non-significant differences are indicated with ‘n.s.’ (see §2; tables 6 and 7 for more information).

**Table 6.** Model result summaries of two GLMMs comparing movements of detritivores and geophages between soil moisture treatment levels (*N* = 117). The model for threshold crossings included a negative binomial family, whereas the model for peak intensity used a log-transformed Gaussian family. Both models included earthworm feeding guild (detritivores versus geophages), soil moisture treatment (dry versus wet) and their interaction as fixed effects, and soil column replicate number as a random intercept. For the earthworm feeding guild treatment, geophages are the reference level, and for soil moisture treatment, wet soil is the reference. See §2 for more details.

	Estimate	SE	*z-*value	*p-*value
**Threshold crossings**				
Intercept	3.006	0.178	16.911	**< 0.001**
Earthworm feeding guild: Detritivore	0.575	0.218	2.643	**0.008**
Moisture level: Dry	–0.623	0.221	–2.817	**0.005**
Earthworm feeding guild: Detritivore * Moisture level: Dry	–0.542	0.318	–1.702	0.089
**Peak intensity**				
Intercept	2.436	0.049	50.070	**<0.001**
Earthworm feeding guild: Detritivore	0.115	0.063	1.810	0.071
Moisture level: Dry	0.230	0.063	3.310	**<0.001**
Earthworm feeding guild: Detritivore * Moisture level: Dry	–0.187	0.091	–2.060	**0.039**

**Table 7.** Tukey's post hoc tests with pairwise comparisons of earthworm movement (threshold crossings and peak intensity) between geophages and detritivores in dry and wet soils, as well as pairwise comparisons of the number of earthworm movements (threshold crossings) between earthworm feeding guilds in dry and wet soils. Post hoc tests followed the statistical models listed in table 6.

	Estimate	SE	*z-*value	*p-*value
**Threshold crossings**				
*Geophage vs. Detritivore*				
Moisture level: Dry	–0.033	0.230	–0.145	0.885
Moisture level: Wet	–0.575	0.218	–2.643	**0.008**
*Wet vs. Dry*				
Earthworm feeding guild: Geophage	0.623	0.221	2.817	**0.005**
Earthworm feeding guild: Detritivore	1.165	0.228	5.098	**<0.001**
**Peak intensity**				
*Geophage vs. Detritivore*				
Moisture level: Dry	–0.023	0.065	–0.344	0.731
Moisture level: Wet	–0.230	0.063	–3.308	**0.001**

When comparing detritivores and geophages within dry and wet soils separately at 200 Hz recording frequencies, detritivores had a significantly higher number of threshold crossings and higher peak intensity values than geophages in wet soils, but this difference was not observed in dry soils (figure 4; tables 6 and 7). To ensure that these differences were consistent in other frequency bands, we confirmed that in all five frequencies (125, 160, 200, 250 and 315 Hz) detritivores showed a higher number of threshold crossings and higher peak intensity values than geophages, although this difference was only significant at 200 and 250 Hz for both variables (supplementary material, tables S1 and S2).

## 4. Discussion

In this study, which investigated earthworm movement detection across different soil moisture conditions, we identified and quantified the vibrations emitted by detritivore and geophage earthworms, and compared them between wet and dry soils, as well as between earthworm feeding guilds. Using a geophone, we detected subterranean movements of earthworms best at the 200 Hz frequency band. Within this frequency band, the number of threshold crossings (indicating the frequency of movement) and the peak intensity (indicating the intensity or strength of movements) of earthworm vibrations detected were significantly higher in wet soils compared to dry soils, regardless of the earthworm feeding guild. In wet soils, detritivore earthworms showed more frequent and more intense movements than geophage earthworms.

Across the wide range of frequencies that vibrations were measured, movements of earthworms in this study were best detected at the 200 Hz frequency band. Whether earthworm predators that use mechanoreceptors to detect their prey will also be able to distinguish earthworm movements from background noises at that frequency will, of course, depend on the sensitivity of the biological tools that predators are equipped with. Many shorebird species, which are common earthworm predators, use the mechanoreceptors, called Herbst corpuscles, in their bill tip organ to locate food by detecting vibrations generated by moving prey in the soil [34–36]. When it comes to the frequency of vibrations to which these remote-sensing birds may respond, Gottschaldt [37] reported that Herbst corpuscles are sensitive to vibratory stimuli within frequencies between 50 and 2000 Hz (at least), with an optimal detection range between 100 and 1000 Hz. The sensitivity of mechanoreceptors in birds has been tested experimentally for a few species only. Herbst corpuscles in the legs of Eurasian bullfinches *Pyrrhula pyrrhula* detect vibrations between 100 and 3200 Hz, although they are most sensitive to frequencies between 100 and 800 Hz [38]. Moreover, mechanoreceptors in the bills of ducks and geese demonstrated sensitivity to vibrations ranging from 50 to 2000 Hz, with geese showing the highest sensitivity for vibrations between 300 and 600 Hz [39–41]. The frequency band of 200 Hz at which earthworm movements were best detected in this study suggests a good fit with the capabilities of remote-tactile foraging birds.

We detected fewer earthworm movements in dry soils than in wet soils. As water is one of the most important abiotic variables affecting earthworm activity [4,42], it is likely that movements of earthworms were more restricted in drier soils. Earthworms may go into a resting phase under low soil moisture conditions, which reduces vibration emissions and consequently detections. For instance, geophages can enter diapause by curling up in a ‘knot’ when soil conditions become too dry [1]. However, within the short time frame of our experiment, we do not expect that in the drier soils geophages were forced into diapause, and indeed we did not observe ‘knotted’ individuals after the measurements. We did, however, notice that detritivores took longer to bury themselves in drier than in wetter soils and we found, in total, four dead detritivores in dry but none in wet soils. This suggests that detritivores might have experienced discomfort in moisture-limited soils, potentially resulting in a lower activity rate. Nonetheless, as water also facilitates the transmission of vibrations [16,17], it is important to recognize that the differences in vibrations that we detected between drier and wetter soils may be the result of a combination of different earthworm activity levels and vibration transmission properties. In addition, it is important to keep in mind that some of the detected vibrations may not be actual earthworm movements, but vibrations resulting from earthworms moving litter down the soil profile or litter manipulation near the soil surface. However, as such vibrations will quickly direct a predator to a moving earthworm, we do suspect that such vibrations will lead to an easier detection of earthworm prey by predators.

In wetter soils we detected more movements of detritivores than geophages. Geophages and detritivores have distinct feeding preferences [3] that may lead to different activity levels while foraging. Geophages consume more soil than detritivores, who primarily feed on organic matter that is not fully decomposed [7,8]. This may be explained by soil particles being widely available underground, reducing the need for geophages to move as much as detritivores. Alternatively, detritivores may have foraged closer to the soil surface than geophages. The detritivores used in this study—*L. rubelles* and juveniles of *L. terrestris*—are likely to behave epigeically and may thus have been closer to the geophone than geophages, where their movements were more likely to be detected. However, if that were the case, the same would hold for predators such as probe-foraging birds, highlighting the experiment's relevance to natural conditions.

The differences in earthworm-induced vibrations as a function of feeding guild and soil moisture content, as reported here, may have repercussions on soil functions and the food web. For example, we predict that remote-tactile foraging predators are more likely to detect earthworm movements in wetter than in drier soils, and that in wetter soils, they may detect detritivores more easily than geophages. That may probably be true for flock-foraging black-tailed godwits during the pre-nesting period in grasslands of the Netherlands. Although godwits detected and captured earthworms of both feeding guilds, including geophages that show no surfacing behaviour, they were found to select detritivores over geophages [43]. Moreover, soil moisture has been shown to allow these godwits to still maintain target foraging rates when food abundance decreases, probably as a result of enhanced earthworm detection capacities [44]. Our study therefore contributes to this body of literature, providing evidence that links these findings with each other towards a more complete understanding of such ecological processes at food web scales.

The experimental approach presented in this study highlights the possibility for investigating subterranean earthworm activity. It may inspire future experimental designs assessing soil fauna activity across a wider range of soil contexts and in different species other than the ones presented here. Such studies could illuminate the different functional roles of earthworm species and their sensitivities to depredation under a range of environmental conditions, such as soil moisture [27] and time of day [45].

## Supplementary Material



## Data Availability

Data for this paper are provided in the supplementary material [46].

## References

[1] Edwards CA , Arancon NQ. 2022 Biology and ecology of earthworms. New York, NY: Springer. (doi:10.1007/978-0-387-74943-3)

[2] Capowiez Y , Marchán D, Decaëns T, Hedde M, Bottinelli N. 2024 Let earthworms be functional - definition of new functional groups based on their bioturbation behavior. Soil Biol. Biochem. 188, 109209. (doi:10.1016/j.soilbio.2023.109209)

[3] Bouché M . 1977 Strategies lombriciennes. Ecol. Bull. 25, 122–132.

[4] Lee K . 1985 Earthworms, their ecology and relationships with soils and land use. Melbourne, Australia: Academic Press.

[5] Hendriksen NB . 1990 Leaf litter selection by detritivore and geophagous earthworms. Biol. Fertil. Soils 10, 17–21. (doi:10.1007/BF00336119)

[6] Curry JP , Schmidt O. 2007 The feeding ecology of earthworms – a review. Pedobiologia 50, 463–477. (doi:10.1016/j.pedobi.2006.09.001)

[7] Judas M . 1992 Gut content analysis of earthworms (Lumbricidae) in a beechwood. Soil Biol. Biochem. 24, 1413–1417. (doi:10.1016/0038-0717(92)90126-I)

[8] Neilson R , Boag B. 2003 Feeding preferences of some earthworm species common to upland pastures in Scotland. Pedobiologia 47, 1–8. (doi:10.1078/S0031-4056(04)70173-0)

[9] Blouin M *et al.* 2013 A review of earthworm impact on soil function and ecosystem services: earthworm impact on ecosystem services. Eur. J. Soil Sci. 64, 161–182. (doi:10.1111/ejss.12025)

[10] Görres JH , McCay T. 2024Earthworm role in soil food webs. In Earthworms and ecological processes (eds Y Kooch, Y Kuzyakov), pp. 433–453. Cham, Switzerland: Springer Nature.

[11] Edwards CA , Bohlen PJ. 1996 Biology and ecology of earthworms. London, UK: Chapman & Hall.

[12] Macdonald DW . 1983Predation on earthworms by terrestrial vertebrates. In Earthworm ecology: from Darwin to vermiculture (ed. JE Satchell), pp. 393–414. London, UK: Chapman & Hall.

[13] du Toit CJ , Chinsamy A, Cunningham SJ. 2024 Good vibrations: remote-tactile foraging success of wading birds is positively affected by the water content of substrates they forage in. J. Avian Biol. 2024, e03243. (doi:10.1111/jav.03243)

[14] Gerritsen AFC , Meiboom A. 1985 The role of touch in prey density estimation by *Calidris* *alba*. Neth. J. Zool. 36, 530–561. (doi:10.1163/002829686X00216)

[15] Stroud JL , Kalkowski MK, Hassall KL, Treadway M, Fannon J, Keith A, Ruiz S, Attenborough K. 2025 The use of ecoacoustics to monitor soil ecology: a critical review with reference to earthworms. Eur. J. Soil Sci. 76, e70229. (doi:10.1111/ejss.70229)

[16] Biot MA . 1956 Theory of propagation of elastic waves in a fluid-saturated porous solid. II. Higher frequency range. J. Acoust. Soc. Am. 28, 179–191. (doi:10.1121/1.1908241)

[17] Biot MA . 1962 Mechanics of deformation and acoustic propagation in porous media. J. Appl. Phys. 33, 1482–1498. (doi:10.1063/1.1728759)

[18] Capowiez Y , Renault P, Belzunces L. 2001 Three-dimensional trajectories of ^60^Co-labelled earthworms in artificial cores of soil. Eur. J. Soil Sci. 52, 365–375. (doi:10.1046/j.1365-2389.2001.00384.x)

[19] Bastardie F , Capowiez Y, Cluzeau D. 2003 Burrowing behaviour of radio-labelled earthworms revealed by analysis of 3D-trajectories in artificial soil cores. Pedobiologia 47, 554–559. (doi:10.1078/0031-4056-00228)

[20] Capowiez Y , Gilbert F, Vallat A, Poggiale J-C, Bonzom J-M. 2021 Depth distribution of soil organic matter and burrowing activity of earthworms—mesocosm study using X-ray tomography and luminophores. Biol. Fertil. Soils 57, 337–346. (doi:10.1007/s00374-020-01536-y)

[21] Bar-Ziv M , Szklowin D, Pearlson O, Vortman Y. 2025 A reproducible model for magnetosensitivity: earthworms in transparent soil reduce their cumulative movement in an extremely weak magnetic field. Biol. Lett. 21, 20250213. (doi:10.1098/rsbl.2025.0213) PMC12503936 41056991

[22] Lacoste M , Ruiz S, Or D. 2018 Listening to earthworms burrowing and roots growing - acoustic signatures of soil biological activity. Sci. Rep. 8, 10236. (doi:10.1038/s41598-018-28582-9) PMC6035217 29980792

[23] Keen SC , Wackett AA, Willenbring JK, Yoo K, Jonsson H, Clow T, Klaminder J. 2022 Non-native species change the tune of tundra soils: novel access to soundscapes of the Arctic earthworm invasion. Sci. Total Environ. 838, 155976. (doi:10.1016/j.scitotenv.2022.155976) 35618134

[24] Singh J , Schädler M, Demetrio W, Brown GG, Eisenhauer N. 2019 Climate change effects on earthworms - a review. Soil Org. 91, 114–138. (doi:10.25674/SO91ISS3PP114) PMC6944501 31908681

[25] Oshima T , Hiraguri Y, Okuzono T. 2015 Distinct effects of moisture and air contents on acoustic properties of sandy soil. J. Acoust. Soc. Am. 138, EL258–EL263. (doi:10.1121/1.4929736) 26428823

[26] Groen NM , Kentie R, Goeij PD, Verheijen B, Hooijmeijer JCEW, Piersma T. 2012 A modern landscape ecology of black-tailed godwits: habitat selection in southwest Friesland, The Netherlands. Ardea 100, 19–28. (doi:10.5253/078.100.0105)

[27] Onrust J , Wymenga E, Piersma T, Olff H. 2019 Earthworm activity and availability for meadow birds is restricted in intensively managed grasslands. J. Appl. Ecol. 56, 1333–1342. (doi:10.1111/1365-2664.13356)

[28] Baltzer R . 1956 Die Regenwürmer Westfalens: eine tiergeographische, oekologische und sinnesphysiologische untersuchung. Zool. Jahresber. 84, 355–414.

[29] Wageningen Environmental Research . *Bodemkaart* *van* *nederland*. https://bodemdata.nl/(accessed 1 March 2023).

[30] van Rhee J . 1970 De regenwormen (Lumbricidae) van Nederland. Wetensch. Meded. KNNV 84, 1–24.

[31] R Core Team. 2022 R: a language and environment for statistical computing. Foundation for Statistical Computing, Vienna. https://www.R-project.org/.

[32] Brooks ME , Kristensen K, Benthem KJV, Magnusson A, Berg CW, Nielsen A, Skaug HJ, Mächler M, Bolker BM. 2017 glmmTMB balances speed and flexibility among packages for zero-inflated generalized linear mixed modeling. R J. 9, 378–400. (doi:10.32614/RJ-2017-066)

[33] Lenth R , Singmann H, Love J, Buerkner P, Herve M. 2018 Package “emmeans”. R Package version 4.0-3. https://CRAN.R-project.org/package%3Demmeans.

[34] Bolze G . 1968 Anordnung und bau der herbstchen körperchen in limicolenschnäbeln im zusammenhang mit der nahrungsfindung. Zool. Anz. 181, 313–355.

[35] Cunningham SJ , Castro I, Potter MA. 2009 The relative importance of olfaction and remote touch in prey detection by North Island brown kiwis. Anim. Behav. 78, 899–905. (doi:10.1016/j.anbehav.2009.07.015)

[36] Cunningham SJ , Alley MR, Castro I, Potter MA, Cunningham M, Pyne MJ. 2010 Bill morphology of Ibises suggests a remote-tactile sensory system for prey detection. Auk 127, 308–316. (doi:10.1525/auk.2009.09117)

[37] Gottschaldt K-M . 1985Structure and function of avian somatosensory receptors. In Form and function in birds (eds A King, J McLelland), pp. 375–461. London, UK: Academic Press.

[38] Schwartzkopff J . 1949 Über den zusammenhang von gehör und vibrationssinn bei vögeln. Experientia 5, 159–161. (doi:10.1007/BF02174430) 18120740

[39] Gregory JE . 1973 An electrophysiological investigation of the receptor apparatus of the duck's bill. J. Physiol. 229, 151–164. (doi:10.1113/jphysiol.1973.sp010132) PMC1350217 4689962

[40] Gottschaldt K-M , Lausmann S. 1974 Mechanoreceptors and their properties in the beak skin of geese *(**Anser* *anser**)*. Brain Res. 65, 510–515. (doi:10.1016/0006-8993(74)90239-X) 4414616

[41] Schneider ER , Gracheva EO, Bagriantsev SN. 2016 Evolutionary specialization of tactile perception in vertebrates. Physiology 31, 193–200. (doi:10.1152/physiol.00036.2015) PMC5005274 27053733

[42] Daugbjerg P . 1988 Temperature and moisture preferences of three earthworm species (Oligochaeta, Lumbricidae). Pedobiologia 32, 57–64. (doi:10.1016/S0031-4056(23)00216-0)

[43] Veenstra R , Onrust J, Verkuil Y, Potkamp G, Berg MP, Henriques M, Piersma T. 2026 In agricultural grassland soils black-tailed godwits select detritivore earthworms. Under review in Ardea.

[44] Veenstra R , Henriques M, Keuning T, Onrust J, Berg MP, Piersma T. 2026 Free-living black-tailed godwits maintain constant intake rates across varying grassland habitat conditions by adjusting their foraging behaviour. IBIS 168, 876–889. (doi:10.1111/ibi.70031)

[45] Onrust J , Piersma T. 2017 The hungry worm feeds the bird. Ardea 105, 153–161. (doi:10.5253/arde.v105i2.a4)

[46] Veenstra R , Halfwerk W, Henriques M, van Hal J, Berg MP, Piersma T. 2026 Supplementary material from: Soil moisture enhances the vibrations induced by moving earthworms, especially of detritivores. Figshare. (doi:10.6084/m9.figshare.c.8683858)

