# Supplementary material for: Soil moisture enhances the vibrations induced by moving earthworms, especially of detritivores
Source: R Soc Open Sci. 2026 Oct 21;13(10):260667. doi: 10.1098/rsos.260667 (PMC13682886; doi:10.1098/rsos.260667)
Supplement: Supplementary file 1 [file rsos.260667.zip › rsos.260667/rsos260667_si_001.docx]

**Supplementary Material**

**From:** Soil moisture enhances the vibrations induced by moving earthworms, especially of detritivores

**Figure S1.** Comparison between the mean of vibration measurements (y-axis, in dB) in columns with and without earthworms, in both dry and wet soils (x-axis), for five frequency bands (vertical panels, 125, 160, 200, 250 and 315 Hz).

**Figure S2.** Comparison between the variance of vibration measurements (y-axis, in dB) in columns with and without earthworms, in both dry and wet soils (x-axis), for five frequency bands (vertical panels, 125, 160, 200, 250 and 315 Hz).

**Figure S3.** Waveforms of vibrations recorded at five 1/3 octave frequency bands (panels) in wet soil columns with detritivore earthworms. Time is on the x-axis, and the vibrations in dB on the y-axis. The green horizontal line marks the threshold calculated for each specific frequency band, used to obtain the variable “threshold crossings”. Thresholds were defined for each measurement by calculating the measurement’s median and adding the mean variance of nine control measurements with the same soil moisture treatment level. These control measurements were taken during quiet evenings in the laboratory building, with the minimum background noise possible. Red points represent each event for in which the vibration levels crossed the threshold.

**Figure S4.** Waveforms of vibrations recorded at five 1/3 octave frequency bands (panels) in wet soil columns without earthworms. Time is on the x-axis, and the vibrations in dB on the y-axis. The green horizontal line marks the threshold calculated for each specific frequency band, used to obtain the variable ‘threshold crossings’. Thresholds were defined for each measurement by calculating the measurement’s median and adding the mean variance of nine control measurements with the same soil moisture treatment level. These control measurements were taken during quiet evenings in the laboratory building, with the minimum background noise possible. Red points represent each event for in which the vibration levels crossed the threshold.

**Figure S5.** Waveforms of vibrations recorded at five 1/3 octave frequency bands (panels) in wet soil columns with earthworms. Time is on the x-axis, and the vibrations in dB on the y-axis. The lowest horizontal green line marks the mean dB of the vibrations, whereas the upper horizontal green line represents the mean dB of the 1% highest vibrations values. The variable ‘peak intensity’ then represents the difference (in dB) between the mean peak values and the mean of the total measurement.

**Figure S6.** Waveforms of vibrations recorded at five 1/3 octave frequency bands (panels) in wet soil columns without earthworms. Time is on the x-axis, and the vibrations in dB on the y-axis. The lowest horizontal green line marks the mean dB of the vibrations, whereas the upper horizontal green line represents the mean dB of the 1% highest vibrations values. The variable ‘peak intensity’ then represents the difference (in dB) between the mean peak values and the mean of the total measurement.

**Table S1.** Model result summaries of two Generalized Linear Mixed Models testing whether detritivore and geophage earthworm movements – threshold crossings and peak intensity – are best detected in wet soils at different frequencies (N = 300). The model for threshold crossings included a negative binomial family, whereas the model for peak intensity used a log-transformed gaussian family. Both models included earthworm ecological group (detritivores vs. geophages), frequency band (125, 160, 200, 250 and 315 Hz) and their interaction as fixed effects, and soil column replicate number as random intercept. For earthworm ecological group, columns without earthworms are the reference level, and for frequency band, the 125 Hz band is the reference.

|  |  | Estimate | SE | z-value | p-value |
| --- | --- | --- | --- | --- | --- |
| **Threshold crossings** |  |  |  |  |  |
| Intercept |  | 2.204 | 0.131 | 16.838 | **<0.001** |
| Earthworm ecological group: Detritivores |  | 0.364 | 0.166 | 2.192 | **0.028** |
| Frequency: |  |  |  |  |  |
| 160 Hz |  | 3.312 | 0.158 | 20.923 | **<0.001** |
| 200 Hz |  | 0.811 | 0.163 | 4.964 | **<0.001** |
| 250 Hz |  | 0.408 | 0.166 | 2.46 | **0.014** |
| 315 Hz |  | 3.628 | 0.158 | 22.95 | **<0.001** |
| Earthworm ecological group * Frequency: |  |  |  |  |  |
| Detritivores: 160 Hz |  | -0.223 | 0.221 | -1.006 | 0.314 |
| Detritivores: 200 Hz |  | 0.181 | 0.227 | 0.799 | 0.424 |
| Detritivores: 250 Hz |  | 0.439 | 0.229 | 1.916 | 0.055 |
| Detritivores: 315 Hz |  | -0.284 | 0.221 | -1.284 | 0.199 |
|  |  |  |  |  |  |
| **Peak intensity** |  |  |  |  |  |
| Intercept |  | 2.503 | 0.052 | 48.525 | **<0.001** |
| Earthworm presence: With earthworms |  | 0.092 | 0.062 | 1.486 | 0.137 |
| Frequency: |  |  |  |  |  |
| 160 Hz |  | -1.116 | 0.062 | -17.988 | **<0.001** |
| 200 Hz |  | -0.067 | 0.062 | -1.079 | 0.280 |
| 250 Hz |  | -0.524 | 0.062 | -8.442 | **<0.001** |
| 315 Hz |  | -1.304 | 0.062 | -21.025 | **<0.001** |
| Earthworm presence * Frequency: |  |  |  |  |  |
| With earthworms : 160 Hz |  | 0.161 | 0.088 | 1.835 | 0.066 |
| With earthworms : 200 Hz |  | 0.118 | 0.088 | 1.34 | 0.180 |
| With earthworms : 250 Hz |  | 0.181 | 0.088 | 2.059 | **0.039** |
| With earthworms : 315 Hz |  | 0.096 | 0.088 | 1.093 | 0.274 |

**Table S2.** Tukey post-hoc tests with pairwise comparisons of threshold crossings and peak intensity values in wet soils at five frequency bands between columns with detritivore and geophage earthworms. Post-hoc tests were conducted following the statistical models presented in Table S2.

|  |  | Estimate | SE | z-value | p-value |
| --- | --- | --- | --- | --- | --- |
| **Threshold crossings** |  |  |  |  |  |
| Geophages vs Detritivores |  |  |  |  |  |
| 125 Hz |  | -0.364 | 0.166 | -2.192 | **0.028** |
| 160 Hz |  | -0.141 | 0.146 | -0.967 | 0.334 |
| 200 Hz |  | -0.545 | 0.155 | -3.526 | **<0.001** |
| 250 Hz |  | -0.803 | 0.158 | -5.071 | **<0.001** |
| 315 Hz |  | -0.080 | 0.146 | -0.548 | 0.584 |
|  |  |  |  |  |  |
| **Peak intensity** |  |  |  |  |  |
| Geophages vs Detritivores |  |  |  |  |  |
| 125 Hz |  | -0.092 | 0.062 | -1.485 | 0.138 |
| 160 Hz |  | -0.253 | 0.062 | -4.081 | **<0.001** |
| 200 Hz |  | -0.210 | 0.062 | -3.381 | **<0.001** |
| 250 Hz |  | -0.273 | 0.062 | -4.398 | **<0.001** |
| 315 Hz |  | -0.188 | 0.062 | -3.032 | **0.003** |
